# *α*-Solanine induces ROS-mediated autophagy through activation of endoplasmic reticulum stress and inhibition of Akt/mTOR pathway

**DOI:** 10.1038/cddis.2015.219

**Published:** 2015-08-27

**Authors:** M Hasanain, A Bhattacharjee, P Pandey, R Ashraf, N Singh, S Sharma, A L Vishwakarma, D Datta, K Mitra, J Sarkar

**Affiliations:** 1 Biochemistry Division, CSIR-Central Drug Research Institute, Lucknow, India; 2 Electron Microscopy Unit, CSIR-Central Drug Research Institute, Lucknow, India; 3 Sophisticated Analytical Instruments Facilities, CSIR-Central Drug Research Institute, Lucknow, India; 4 Academy of Scientific and Innovative Research, Chennai, India

## Abstract

*α*-Solanine is a glycoalkaloid found in species of the nightshade family including potato. It was primarily reported to have toxic effects in humans. However, there is a growing body of literature demonstrating *in vitro* and *in vivo* anticancer activity of *α*-solanine. Most of these studies have shown activation of apoptosis as the underlying mechanism in antitumor activity of *α*-solanine. In this study, we report *α*-solanine as a potential inducer of autophagy, which may act synergistically or in parallel with apoptosis to exert its cytotoxic effect. Induction of autophagy was demonstrated by several assays including electron microscopy, immunoblotting of autophagy markers and immunofluorescence for LC3 (microtubule-associated protein 1 (MAP1) light chain-3) puncta. *α*-Solanine-induced autophagic flux was demonstrated by additionally enhanced – turnover of LC3-II and – accumulation of LC3-specific puncta after co-incubation of cells with either of the autophagolysosome inhibitors – chloroquine and – bafilomycin A1. We also demonstrated *α*-solanine-induced oxidative damage in regulating autophagy where pre-incubation of cells with reactive oxygen species (ROS) scavenger resulted in suppression of CM-H_2_DCFDA (5 (and 6)-chloromethyl-2′,7′-dichlorodihydrofluorescein diacetate acetyl ester) fluorescence as well as decrease in LC3-II turnover. *α*-Solanine treatment caused an increase in the expression of endoplasmic reticulum (ER) stress proteins (BiP, activating transcription factor 6 (ATF6), X-box-binding protein 1, PERK, inositol-requiring transmembrane kinase/endonuclease 1, ATF4 and CCAAT-enhancer-binding protein (C/EBP)-homologous protein) suggesting activation of unfolded protein response pathway. Moreover, we found downregulation of phosphorylated Akt (Thr^308^ and Ser^473^), mammalian target of rapamycin (mTOR; Ser^2448^ and Ser^2481^) and 4E-BP1 (Thr^37/46^) by *α*-solanine implying suppression of the Akt/mTOR pathway. Collectively, our results signify that *α*-solanine induces autophagy to exert anti-proliferative activity by triggering ER stress and inhibiting Akt/mTOR signaling pathway.

Glycoalkaloids (GAs) are secondary plant metabolites produced as natural toxins in order to protect the plants from hostile environments such as cold stress, insects, phytopathogen attacks and vertebrate feeding. Although GAs are found in several fruits and vegetables, potato (*Solanum tuberosum* L.) is the major source of GAs in human diet.^1^ GAs are highly concentrated in the flower and sprouts of potato and also found at relatively low amount in the tuber.^2^ Although there are several reports on human poisoning because of potato alkaloids,^3, 4, 5^ the available data on toxic effect of GA in human health is still incomplete.^6^ *α*-Chaconine and *α*-solanine are two major constituents (95%) of total GAs in potato.^1^ The ratio of *α*-chaconine in potatoes is three times higher than that of *α*-solanine.^7^ *α*-Chaconine has also been reported to be more toxic than *α*-solanine.^8^ Traditionally held view is that human consumption of potato GAs at 3–6 mg/kg body weight is lethal and >1–3 mg/kg body weight has toxic effect of gastrointestinal disturbances and neurological disorders.^6^

*α*-Solanine, a trisaccharide GA, is produced biosynthetically via cholesterol pathway.^7^ The toxic level of *α*-solanine in human diet is not defined yet. In spite of general perception that GAs including *α*-solanine are toxic, they have been shown to produce beneficial effect in human health depending on concentration and condition of use. *α*-Solanine and other GAs showed anti-allergic,^9^ anti-pyretic,^10^ anti-inflammatory,^10, 11^ anti-diabetic^12^ and antibiotic activity against pathogenic bacteria,^13, 14^ viruses,^15, 16^ fungi^17^ and protozoa.^18^ Although there are several publications on *in vitro* anti-proliferative activity of *α*-solanine on various human cancer cell lines,^19, 20, 21, 22, 23^ its *in vivo* therapeutic efficacy against mouse model of human cancer has been reported recently.^22, 23, 24^ Most of these reports have shown that the anticancer activity of *α*-solanine is mediated through induction of apoptosis.

Macroautophagy (referred to as autophagy hereafter in this article) is an evolutionarily conserved cellular process of self-digestion wherein cellular proteins and organelles are degraded by lysosomal enzymes in response to intracellular and extracellular stresses, such as starvation.^25^ In the absence of stress, autophagy occurs at low basal level to maintain cellular homeostasis by degrading intracellular damaged proteins and organelles.^26^ Several human diseases, including cancer, are found to be associated with malfunctioning of the autophagic process.^27, 28^ Accumulating evidences have established that several chemotherapeutic agents trigger autophagy to kill cancer cells.^29, 30, 31^ Autophagy has also been considered as a therapeutic target in cancer cells that are resistant to anticancer drugs.^32^ Recently, it has been demonstrated that aqueous extract of *Solanum nigrum* leaves, which is a rich source of solanine induces autophagy in human colorectal cancer cells.^33^ Given these observations together with previous reports on anticancer activity of *α*-solanine, we designed the study to decipher the role of autophagy and its underlying mechanism in *α*-solanine-mediated cancer cell death.

Here, we demonstrate for the first time that *α*-solanine triggers autophagy-associated cell death in human cancer cells. We further show stimulation of endoplasmic reticulum (ER) stress and inhibition of AKT-mammalian target of rapamycin (mTOR) signaling pathway by *α*-solanine, which may have a vital role in inducing autophagy. Our findings provide the groundwork for planning future studies on anticancer activity of *α*-solanine.

## Results

### *α*-Solanine induces autophagy in human cancer cells

**Figure 1:**
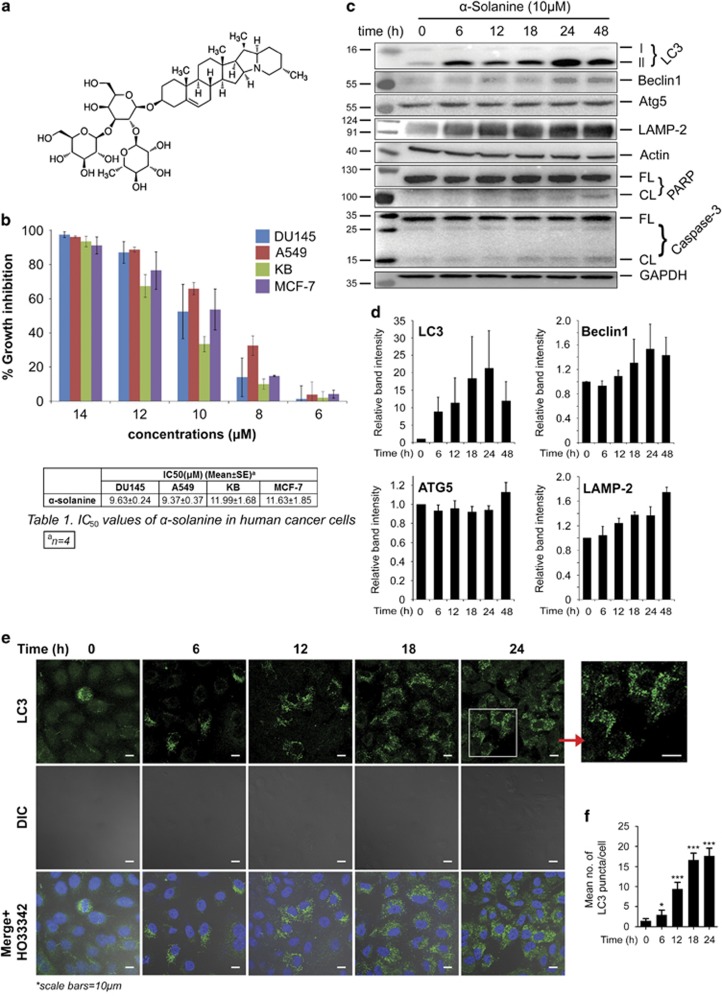
*α*-Solanine-induced autophagy in A549 cells. (**a**) Chemical structure of *α*-solanine. (**b**) Cytotoxic effect of *α*-solanine. Cells were treated with *α*-solanine for 48 h at indicated concentration and cell viability was measured by SRB assay. (**c**) A549 cells were treated with 10 *μ*M *α*-solanine for indicated time points. Cell lysates were analyzed by immunoblotting for autophagic and apoptotic markers. (CL, cleaved; FL, full length). (**d**) Bar graphs representing densitometric quantification of the western blot data (mean±S.E.) of three independent experiments. (**e**) A549 cells were treated with 10 *μ*M *α*-solanine and were fixed at different time points. Cells were then reacted with anti-LC3 antibody and were analyzed by confocal microscopy after incubation with Alexafluor 488 tagged anti-rabbit IgG. (**f**) Bar graph representing average number of typical LC3 puncta/cell. Data are means±S.E. from minimum 25 cells for each experiment; *P=0.2482, ***P<0.0001, compared with untreated control

We first assessed *in vitro* anti-proliferative activity of *α*-solanine (Figure 1a) by sulforhodamine B (SRB) assay on a panel of human cancer cell lines comprising A549, MCF-7, DU145 and KB. Incubation of cells with *α*-solanine for 48 h resulted in reduction in viability with IC_50_ values ~10 *μ*M in all cell lines (Figure 1b, Table 1). The least differences in the IC_50_ values among different human cancer cells suggest broad spectrum anti-proliferative activity of *α*-solanine. As a result of A549 being most sensitive, this cell line was selected for most of the subsequent studies. As autophagy and apoptosis are two major modes of cell death in response to cellular stress, we investigated the ability of *α*-solanine to activate these cellular processes. Induction of autophagy was examined by determining the level of LC3 (microtubule-associated protein 1 (MAP1) light chain-3) by western blot assay. LC3, a subunit of microtubule-associated proteins 1A and 1B (termed MAP1LC3),^34^ is a mammalian homolog of yeast Atg8.^35^ During autophagy, LC3 is cleaved at carboxy terminus by Atg4 to form LC3-I, which is eventually converted to LC3-II through lipidation by Atg7 and Atg3. Hence, LC3-II is widely used as an indicator of autophagy.^36^ In this study, A549 cells were treated with *α*-solanine at IC_50_ concentration for different time intervals and LC3-II expression was measured by western blot assay. As shown in Figures 1c and d, *α*-solanine treatment resulted in time-dependent conversion of LC3-I to LC3-II where highest expression of LC3-II was observed at 24 h. On the contrary, maximum cleavage of poly (ADP-ribose) polymerase (PARP) and caspase-3 was observed at 48 h post-exposure to *α*-solanine denoting induction of apoptosis. Subsequently, cells were treated with *α*-solanine up to 24 h in subsequent studies on autophagy. In accordance with increased LC3-II expression, other key autophagy regulating proteins such as Beclin 1, lysosome-associated membrane protein 2 (LAMP-2) and autophagy-related 5 (ATG5) were also increased in time-dependent manner following *α*-solanine treatment (Figures 1c and d).

Conjugation with lipids allows LC3 to be relocated toward autophagic vesicles and subsequently become associated with autophagosomal membrane.^35^ Therefore, monitoring the changes from a diffused pattern of LC3 to accumulation of LC3 puncta in cell cytoplasm is another way of detecting autophagosomes by fluorescence microscopy.^37^ Here, cellular distribution of LC3 in A549 cells was examined by immunofluoroscence before and after exposure to *α*-solanine. As can be seen in Figure 1e, *α*-solanine treatment resulted in accumulation of LC3-specific puncta in time-dependent manner. The changes were statistically significant from 12 h onward post-exposure to *α*-solanine in comparison with untreated controls (Figure 1f). We also sought to investigate if *α*-solanine induces autophagy in other human cancer cell lines as well. As shown in Supplementary Figure S1A, *α*-solanine enhanced expression of Beclin 1 in all the cell lines. Correspondingly, LC3B-II was found to be upregulated after *α*-solanine treatment in all the cell lines except DU145 where no expression of LC3B-II was noticed. *α*-Solanine treatment of DU145 did not result in detectable level of ATG5 as well (data not shown), whereas it induced PARP cleavage in all the cell lines (Supplementary Figure S1B). In addition, treatment of *α*-solanine to a stable C33A cell line expressing GFP-tagged LC3 (C33A-GFP-LC3) resulted in marked accumulation of green fluorescent dots than untreated controls denoting induction of autophagy (Supplementary Figure S1C).

**Figure 2:**
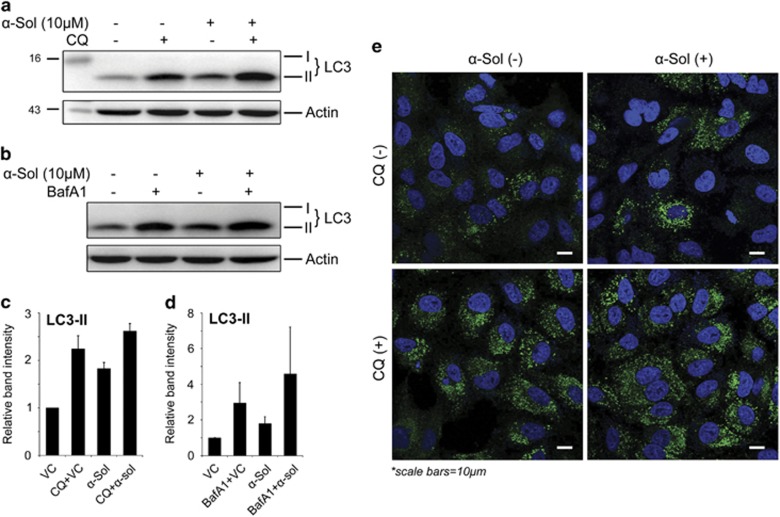
*α*-Solanine-induced autophagic flux. A549 cells were treated with *α*-solanine for 24 h without or with 5 *μ*M CQ (2 h pre-incubation). Cellular LC3 was analyzed by western blot assay (**a** and **c**) and by confocal microscopy (**e**). (**b** and **d**) Western blot analysis of A549 cell lysates treated with *α*-solanine (10 *μ*M, 24 h) in presence or absence of 100 nM BafA1 (2 h pre-incubation) using anti-LC3 antibody

**Figure 3:**
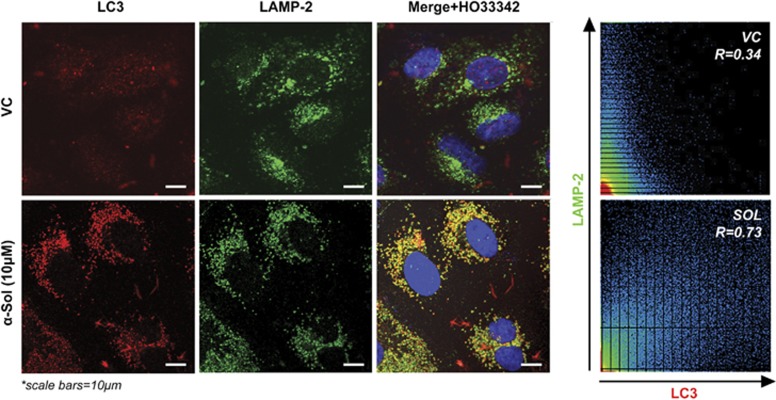
*α*-Solanine treatment caused autophagosome–lysosome fusion. A549 cells were treated with *α*-solanine at 10 *μ*M for 24 h and immunostained with anti-LC3 (marker for autophagosome) and anti-LAMP2 (marker for lysosome) antibodies. Fluorescently labeled cells were analyzed under confocal microscope

*α*-Solanine-induced autophagic flux was further investigated in the presence and absence of autophagosome–lysosome fusion inhibitors, bafilomycin A1 (BafA1) and chloroquine (CQ). As expected, 2-h pretreatment with 100 nM BafA1 or 5 *μ*M CQ alone resulted in increased LC3B-II level, whereas combination of BafA1/CQ and *α*-solanine caused additionally enhanced turnover of LC3-II (Figures 2a and d). Similar results were obtained in microscopic analysis where additionally enhanced accumulation of LC3 puncta was seen after 24-h treatment of *α*-solanine in cells pre-incubated with CQ (Figure 2e). To further confirm *α*-solanine-induced autophagic flux, fusion of autophagosome (LC3 positive) with lysosome (LAMP-2 positive) was investigated by confocal microscopy (Figure 3) where significant colocalization of the two compartments was observed post-treatment. Finally, we inhibited autophagy through siRNA-mediated downregulation of Beclin 1. The knock down efficiency of siRNA was confirmed by immunoblotting showing significant reduction in Beclin1 expression after *α*-solanine treatment (Supplementary Figure S1D). Accordingly, expression of LC3B-II (Supplementary Figure S1D) and ATG5 (data not shown) were also downregulated in siRNA-transfected cells. Although these results imply inhibition of autophagy, they also corroborate induction of autophagic flux by *α*-solanine.

In view of the role of autophagy in promoting either cell survival or death, we were interested to see how autophagy contributes to the cytotoxic effect of *α*-solanine. Thus, A549 cells were incubated with *α*-solanine and doxorubicin or either of them for 24 h. Scanning electron microscopic (SEM) analysis revealed marked increase in apoptotic population in *α*-solanine and doxorubicin-treated cells with characteristic apoptotic bodies and membrane blebbing than other treatment groups (Supplementary Figure S2A). Similarly, we also saw significant reduction in viability of cells after co-treatment with *α*-solanine and doxorubicin than treatment with either of them (Supplementary Figures S2B and C).

### Ultrastructural alterations induced by *α*-solanine

**Figure 4:**
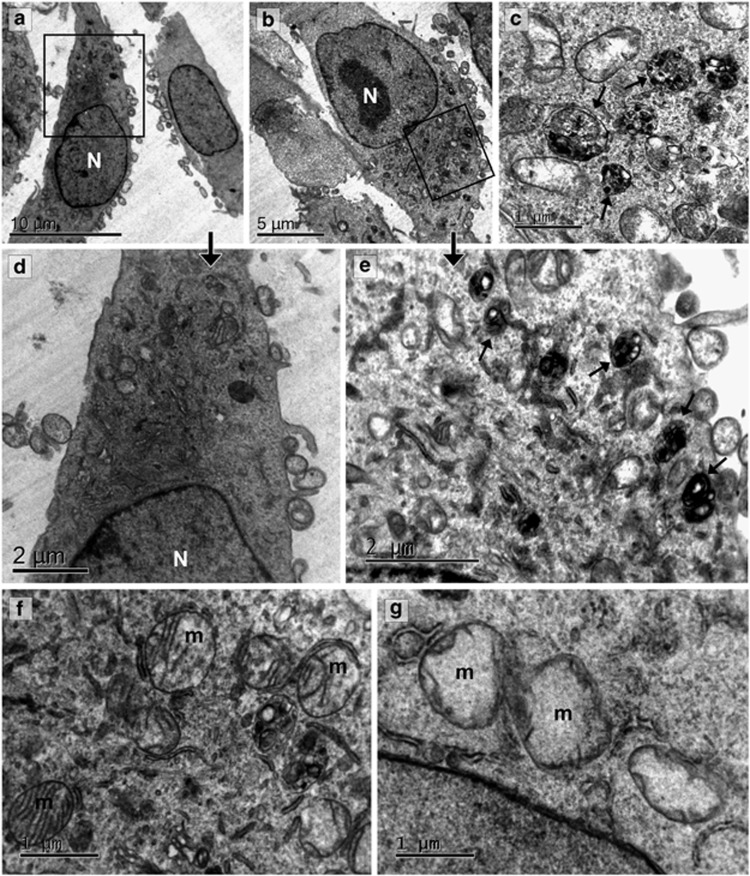
Ultrastructural study of A549 cells treated with *α*-solanine. Representative electron microscopic images of A549 cells treated without (**a** and **d**) or with (**b**, **c** and **e**) *α*-solanine (10 *μ*M, 24 h) showing accumulation of autophagic vacuoles (marked with arrows) in treated cells. (**f**) Electron micrograph showing cristae and electron-dense mitochondrial matrix in vehicle-treated cells. (**g**) Lack of mitochondrial electron density and cristae was evident after *α*-solanine treatment

Subcellular alterations in A549 cells induced by *α*-solanine were analyzed by TEM. Control cells showed normal ultrastructure with cytoplasm containing structurally intact organelles like Golgi vesicles, rough ER and mitochondria consisting of well-defined cristae and electron-dense mitochondrial matrix (Figures 4a, d and f). The cells were spindle shaped and the nuclei contained evenly distributed chromatin (Figure 4a). Cells treated with 10 *μ*M *α*-solanine for 24 h showed abundant autophagosomes, amphisomes and autolysosomes in various stages of maturation (Figures 4b, c and e). Mitochondria appeared less electron-dense with disruption in mitochondrial ultrastructure and loss of cristae (Figure 4g) Cells exhibited swollen morphology with no visible change in the nucleus or the plasma membrane. Significant ER swelling was observed in treated cells indicating ER stress (Supplementary Figure S3).

### *α*-Solanine-induced autophagy is mediated through accumulation of intracellular ROS

**Figure 5:**
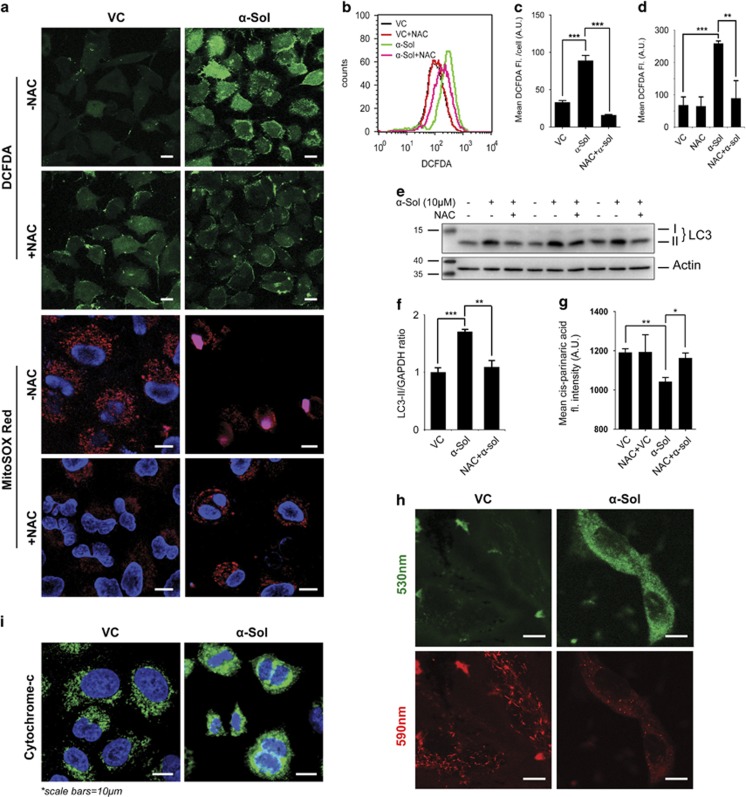
*α*-Solanine-induced intracellular ROS to trigger autophagy. (**a**) A549 cells were treated with 10 *μ*M *α*-solanine in presence or absence of 5 mM NAC. Cells were then stained with DCFDA and MitoSOX Red dye before being examined under confocal microscope. (**b**) Flow cytometric analysis of DCFDA-stained A549 cells for ROS accumulation. (**c**) Mean fluorescence intensities of at least 25 microscopically examined cells for each treatment were plotted and analyzed statistically (**c**); ***P<0.005. (**d**) Mean DCFDA fluorescence of *α*-solanine-treated A549 cells was analyzed by flow cytometry before and after exposure to NAC. (**e**) *α*-Solanine-treated cell lysates (with or without pre-incubation with NAC) were analyzed by western blot assay using specific antibodies. (**f**) Densitometric analysis of LC3-II levels relative to GAPDH. **P<0.05; ***P<0.005. (**g**) Increased lipid peroxidation in *α*-solanine-treated cells. (**h**) Effect of *α*-solanine on MMP of A549 cells. Cells were treated with 10 *μ*M *α*-solanine, stained with 2 *μ*M JC1 and examined under microscope. (**i**) *α*-Solanine caused release of cytochrome c from mitochondria. A549 cells were treated with 10 *μ*M *α*-solanine for 24 h, immunostained with cytochrome c and examined under confocal microscope

It is now well appreciated that reactive oxygen species (ROS) has an essential role in the autophagic process.^38^ Mitochondria are the major source of ROS inside the cells. Our electron microscopic study revealed damage of mitochondria in *α*-solanine-treated cells, which was evident as severe mitochondrial swelling and loss of cristae network (Figure 4g). Consistent with electron microscopy data, analysis of JC1-stained A549 cells by confocal microscopy revealed loss of mitochondrial membrane potential (Figure 5h) in *α*-solanine-treated cells, which in turn caused release of cytochrome c into the cytosol (Figure 5i). This led us to postulate that *α*-solanine may trigger release of ROS from mitochondria as well which can be an important factor in inducing autophagy. To examine whether ROS is enhanced because of *α*-solanine treatment, cells were stained with CM-H_2_DCFDA (5 (and 6)-chloromethyl-2′,7′-dichlorodihydrofluorescein diacetate acetyl ester) and examined under confocal microscope. As can be seen in Figures 5a and c, *α*-solanine caused an increase in CM-H_2_DCFDA fluorescence of ~63% compared with control, which was scavenged by the antioxidant and ROS scavenger, *N*-acetyl-L-cysteine (NAC), reflecting ~82% decrease in fluorescence. In agreement with microscopic data, analysis of CM-H_2_DCFDA stained A549 cells by flow cytometry revealed significant increase in mean fluorescence of *α*-solanine-treated cells compared with untreated control, which was further decreased upon NAC exposure (Figures 5b and d). We also used MitoSOX Red to monitor mitochondrial superoxide levels in A549 cells. This fluorogenic dye is highly selective for mitochondrial superoxide and is not oxidized by any other ROS or reactive nitrogen species. The oxidation product of the probe becomes highly fluorescent upon binding with nucleic acids as observed in *α*-solanine-treated cells indicating elevated superoxide levels in the mitochondria (Figure 5a). Control cells showed basal levels of mitochondrial superoxide indicated by the fluorescence of the oxidized product upon binding with mitochondrial DNA, whereas NAC pretreatment resulted in decreased fluorescence. *α*-Solanine-mediated upregulation of free radicals was additionally authenticated by lipid peroxidation assay using a naturally fluorescent fatty acid cis-parinaric acid. Here, significant loss of fluorescence intensity was observed after *α*-solanine treatment, which was subsequently restored on pre-incubation with NAC (Figure 5g). Altogether, findings from these experiments signify induction of ROS by *α*-solanine. To further confirm the role of ROS on *α*-solanine-mediated autophagy, the level of LC3-II was examined in presence and absence of NAC after treatment with *α*-solanine. As shown in Figures 5e and f, pretreatment with NAC caused significant decrease in *α*-solanine-mediated enhanced LC3-II level implying regulation of autophagy by ROS.

### ER stress is involved in *α*-solanine-induced autophagy

**Figure 6:**
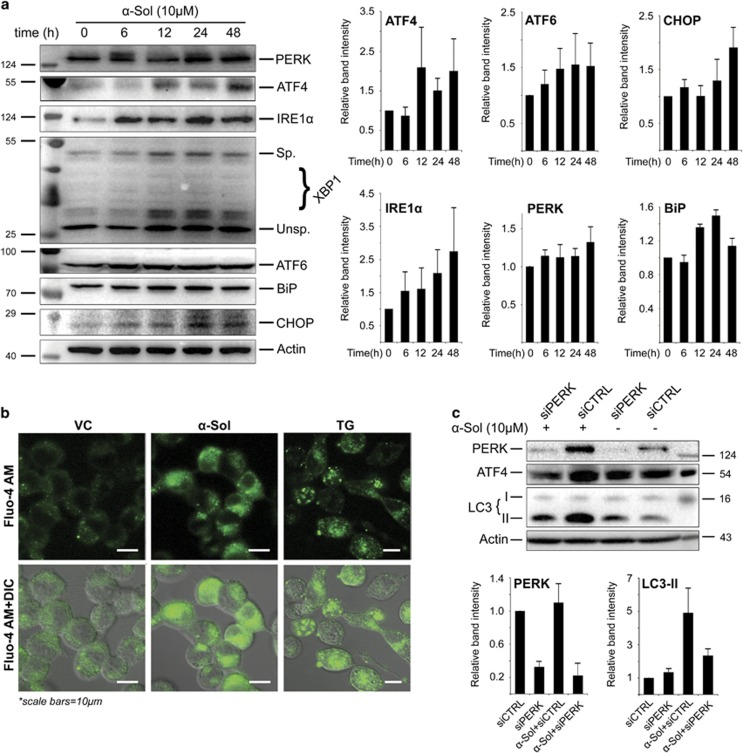
*α*-Solanine activated UPR pathway. (**a**) A549 cells were exposed to 10 *μ*M *α*-solanine and harvested at indicated time points. Cell lysates were subjected to western blot assay to determine activation of UPR pathway and band intensities were quantified. (**b**) ER calcium ion release in *α*-solanine (10 *μ*M, 24 h) treated cells was investigated by fluorescence microscopy after staining with 5 *μ*M Fluo-4AM. (**c**) A549 cells were transfected with non-targeting or PERK siRNA and treated with *α*-solanine for 24 h. The corresponding proteins were evaluated by immunoblotting and band intensities were quantified

To determine the ability of *α*-solanine to induce ER stress, the expression of signaling molecules of unfolded protein response (UPR) pathway was examined by western blotting (Figure 6a). Exposure of A549 cells to *α*-solanine resulted in an increase in the level of ER chaperone, BiP/GRP78. Expression of other transmembrane sensors like inositol-requiring transmembrane kinase/endonuclease 1 (IRE1) and PERK was also elevated in *α*-solanine-treated cells. Correspondingly, *α*-solanine-induced expression of the transcription factors activating transcription factor 6 (ATF6), X-box-binding protein 1 (XBP1) and ATF4, which are activated as an adaptive response during ER stress. CCAAT-enhancer-binding protein (C/EBP)-homologous protein (CHOP)/GADD153, another transcription factor having binding sites for ATF6, XBP1 and ATF4 at its promoter region and mediator of cell death during ER stress, was found to be upregulated after *α*-solanine treatment. Consistent with immunoblot data, ultrastructural study by electron microscopy revealed swelling of the ER lumen with smooth surface in *α*-solanine-treated cells confirming ER stress (Supplementary Figure S3). These results indicate that *α*-solanine is a potent inducer of ER stress and UPR pathway. Taking cues from our observation that *α*-solanine treatment induced ER stress, we investigated calcium efflux from the ER as it is known that early ER stress involves calcium leakage from the ER. Cytosolic calcium levels were monitored using fluorescent calcium indicator Fluo-4AM. Control cells showed low fluorescence indicating a basal level of cytosolic calcium. As evident from Figure 6b, *α*-solanine-treated cells showed significantly increased green fluorescence because of elevated cytosolic calcium levels. A similar effect was observed in Thapsigargin-treated cells, which suggests that *α*-solanine treatment induces calcium efflux from ER stores (major intracellular store for releasable calcium) into the cytosol. Similar observations have been reported earlier^39^ in Panc-1 pancreatic cancer cells with 3,3-diindolylmethane (DIM). Oxidative stress and ROS generation are integral part of ER stress, which have both upstream and downstream roles in the UPR pathway. Although ROS has the potential to induce ER stress, it is produced as a byproduct at the time of disulfide bond formation during protein folding. To determine possible role of ROS in activating UPR pathway, we examined PERK level in presence or absence of NAC after *α*-solanine treatment. As can be seen in Supplementary Figure S4, pre-incubation with NAC resulted in marked downregulation of *α*-solanine-induced PERK level suggesting involvement of ROS in triggering ER stress. To further investigate role of ER stress in *α*-solanine-induced autophagy, cells were transfected with siRNA targeting PERK. Depletion of PERK activity resulted in reduced expression of ATF4 and decreased level of LC3-II in *α*-solanine-treated cells (Figure 6c). Collectively, above findings suggest that activation of UPR pathway by *α*-solanine promotes autophagy.

### *α*-Solanine induces autophagy through inhibition of Akt/mTOR pathway

**Figure 7:**
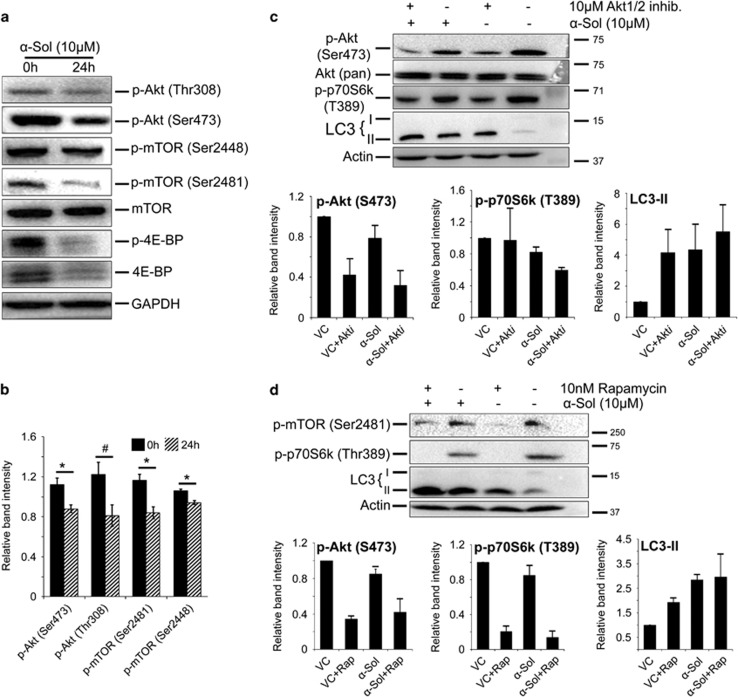
*α*-Solanine inhibited Akt/mTOR signaling. (**a**) A549 cells were analyzed for Akt/mTOR activity by western blot assay using pathway-specific antibodies after incubation with or without *α*-solanine for 24 h. (**b**) Densitometric analysis on band intensity of corresponding proteins relative to loading-control. ^#^*P*<0.05; *P<0.005. A549 cells were treated with *α*-solanine in presence or absence of 10 *μ*M Akt1/2 inhibitor (**c**) and 10 nM rapamycin (**d**). Cell lysates were analyzed by immunoblotting with corresponding antibodies

Previous reports have established Akt/mTOR as a key signaling pathway, which negatively regulates autophagy.^25^ To gain further insight into the molecular mechanism of *α*-solanine-induced autophagy, we investigated expression and post-translational modification of key regulatory proteins of Akt/mTOR pathway. Phosphorylation of Akt at Thr^308^ and Ser^473^ are used as markers of active Akt, which inhibit apoptosis and autophagy. In this study, significant downregulation of p-Akt (Ser^473^and Thr^308^) was observed in *α*-solanine-treated cells suggesting involvement of this pathway in triggering autophagy (Figures 7a and b). mTOR, which acts downstream to Akt in Akt/mTOR pathway, is phosphorylated during growth factor-induced cell signaling and negatively regulates autophagy. Here, significantly reduced level of phosphorylated mTOR (Ser^2448^ and Ser^2481^) was noticed after *α*-solanine treatment, while there seems to be no obvious change in total mTOR level between treated and untreated groups (Figures 7a and b). Similarly, phosphorylation of 4E-BP1 (eukaryotic initiation factor 4E-binding protein 1) at Thr^37/46^, a downstream target of mTORC1, was diminished following *α*-solanine treatment (Figure 7b). Level of total 4E-BP1 was also decreased in *α*-solanine-treated A549 cells in comparison with the untreated control. To confirm regulatory role of Akt/mTOR pathway in *α*-solanine-induced autophagy, cells were treated with Akt1/2 inhibitor and rapamycin and assessed for level of autophagy. As shown in Figure 7c, inhibition of Akt1/2 was accompanied by increased autophagic signaling as indicated by additionally enhanced LC3-II level in *α*-solanine-treated cells. Similarly, mTOR inhibition by rapamycin resulted in decreased phosphorylation of target protein, p70S6K at Thr^389^ and enhanced autophagic effect of *α*-solanine (Figure 7d). Taken together, above results indicate that *α*-solanine suppresses Akt/mTOR kinase activity to induce autophagy.

## Discussion

The pharmacological effect of *α*-solanine in human has long been a matter of debate. Although several investigators have reported this GA to be toxic to humans,^3, 4, 5^ there have been an increasing number of literatures on therapeutic potential of *α*-solanine against several ailments including cancer.^9, 12, 13, 19,20,21,22,23^ Until now, apoptosis was believed to be the crucial factor in inducing cell death by *α*-solanine.^22, 23, 24, 40^ Here, we investigated the ability of *α*-solanine to induce cell death by autophagy and its underlying molecular mechanism. We demonstrate that *α*-solanine triggers both apoptosis and autophagy to mediate cell death. However, induction of autophagy appears to be an early event in A549 cells, where highest conversion of LC3B-II was seen at 24 h, than apoptosis, where maximum cleavage of PARP and caspase-3 was seen at 48 h. In contrast to other cell lines, we could not detect key autophagy markers (LC3 and ATG5) in DU145 cells. This could be due to the defect in autophagy pathway in this cell line because of the absence of functional ATG5.^41^ However, in view of nearly equal sensitivity of all the cell lines including DU145, toward *α*-solanine, it appears that autophagy may not be an essential mechanism in inducing cell death by *α*-solanine. Instead, it may act independently or synergistically with apoptosis to exert the cytotoxic effect of *α*-solanine. Consistent with this hypothesis, we observed an additive effect on cell cytotoxity when the cells were co-treated with *α*-solanine and doxorubicin, a known cytotoxic agent.

We carried out several studies – like conversion of LC3B-I to LC3B-II, quantification of LC3B puncta, expression of ATG5 and Beclin 1 and ultrastructural morphology to demonstrate autophagy in the most susceptible cell line, A549. Nevertheless, increase in cellular level of LC3-II, Beclin 1 and ATG5 does not ensure autophagic proteolysis of cellular proteins and organelles. As increased turnover of LC3-II can occur either in augmented autophagosome formation or because of impaired autophagosome–lysosome fusion.^42^ Similarly, Beclin 1 and ATG5 are not specific markers of autophagy and they are also involved in regulating apoptosis.^43, 44^ To address this concern, we measured autophagic flux through several methods in *α*-solanine-treated A549 cells. RNAi knockdown of Beclin 1 resulted in reduced conversion of LC3B-I to LC3B-II and downregulation of ATG5, implying Beclin 1 has a critical role in *α*-solanine-induced autophagy. Conversely, pre-exposure of A549 cells to autophagosome–lysosome fusion inhibitors BafA1 and CQ resulted in increased LCB-II conversion and rise in LC3B-specific puncta. This may be due to an increased autophagosome accumulation in the cytoplasm collectively by *α*-solanine and blockage of autophagosome clearance by BafA1 or CQ. In addition, we verified *α*-solanine-induced autophagosome–lysosome fusion by electron microscopy and immunoflurescence. Collectively, our data demonstrate that *α*-solanine is indeed able to induce autophagy.

In eukaryotic cell, ER is primarily engaged in folding and maturation of secretary and membrane-associated proteins and the ER homeostasis is precisely regulated by several sensor and regulatory factors. Any perturbation in protein folding leads to activation of a conserved signaling pathway, collectively called as UPR pathway, in order to decrease protein flux as well as to increase protein folding capacity in ER. In addition to its role in initiating apoptotic process, emerging reports are beginning to establish association between ER stress and induction of autophagy.^45, 46, 47^ ER stress is sensed and responded by 3 UPR signal transducers *viz*. IRE1, PERK and ATF6.^48^ These transmembrane sensors remain suppressed in non-stressed cells by binding to a chaperone, BiP/GRP78. Upon ER stress, available pool of BiP is occupied with misfolded proteins, resulting in derepression of these sensors including ATF6, which in turn transactivates BiP.^49^ Here, we observed upregulation of ATF6 along with its target, BiP implying induction of ER stress by *α*-solanine. Our results also demonstrate activation of other arms of UPR pathway by means of induction of IRE1 and PERK together with their effector molecules XBP1 (XBP1s) and ATF4. Splicing of XBP1 enables it to translocate to nucleus where it activates transcription of an array of genes involved in protein folding and degradation to restore ER homeostasis.^48^ Recent evidence suggests that XBP1s triggers autophagy by transactivating Beclin 1.^50^ In view of the ability of ATF6 in transactivating XBP1,^51^ it is also possible that ATF6 may also acts as a mediator in *α*-solanine-induced autophagy. In this study, we observed upregulation of a death-inducing transcription factor, CHOP, following treatment with *α*-solanine. CHOP acts as a junction for the entire ER stress sensor transduced signaling pathways by harboring binding sequences for XBP1, ATF6 and ATF4 at its promoter region.^51, 52^ It promotes autophagy by alleviating Beclin 1 inhibition through downregulation of Bcl-2.^53^ Earlier report on involvement of CHOP in inducing ROS by transactivation of ERO1 ^(ref 54)^ supported with the electron microscopic result of damaged mitochondrial morphology and altered mitochondrial membrane potential, led us to postulate that *α*-solanine may cause oxidative damage to A549 cells. Indeed, we observed significantly increased cellular ROS level after *α*-solanine treatment through several assays as well as reduction in LC3 lipidation when ROS was inhibited by NAC. Increased level of intracellular ROS triggered ER stress, which in turn induced autophagy. It is also likely that enhanced ROS may have inactivated Atg4 and consequently promoted LC3B lipidation to induce autophagy.^38^ Collectively, our data indicate that *α*-solanine triggers UPR pathway and oxidative damage to induce autophagy.

Akt/mTOR pathway has long been implicated in regulating apoptosis and autophagy.^55, 56^ It has recently been shown that *α*-solanine induces apoptosis by blocking Akt/mTOR pathway.^22^ In line with this finding, we also observed downregulation of Akt and mTOR phosphorylation in *α*-solanine-treated cells. Phosphorylation at Thr^308^ and Ser^473^ by upstream kinases is essential for complete activation of Akt in response to growth factor and other extracellular stimuli. Activated Akt in turn relieves mTOR inhibition by phosphorylating its negative regulators – TSC1, TSC2 and PRAS40.^57, 58^ The function of mTOR is also regulated by phosphorylation at Ser^2448^ (by Akt) and at Ser^2481^ (autophosphorylation).^59, 60^ In eukaryotes, mTOR exists in two functionally distinct complexes mTORC1 and mTORC2, depending on its phosphorylation status and the co-factors bound to it. In this study, we observed downregulation in phosphorylation of mTOR at Ser^2448^ and Ser^2881^, which denotes inhibition of both mTORC1 and mTORC2, respectively.^61^ mTORC1 inhibits initiation of autophagy through phosphorylation-dependent suppression of Atg13 and Ulk1/2 kinase.^62, 63, 64^ Conversely, there is no evidence on direct effect of mTORC2 on autophagy. Nonetheless, it activates Akt through phosphorylation at Ser^473 (ref.65)^ and thus negatively regulates autophagy. Our result also revealed downregulation of phosphorylated 4E-BP1 (Thr^37/46^) after *α*-solanine treatment. Given that phosphorylation of 4E-BP1 is promoted by mTORC1, our results confirm inhibition of mTORC1 by *α*-solanine. These results, supported with additional increase in LC3-II level upon inhibition of Akt and mTOR kinase activity, imply that *α*-solanine inhibits Akt/mTOR pathway to induce autophagy.

**Figure 8:**
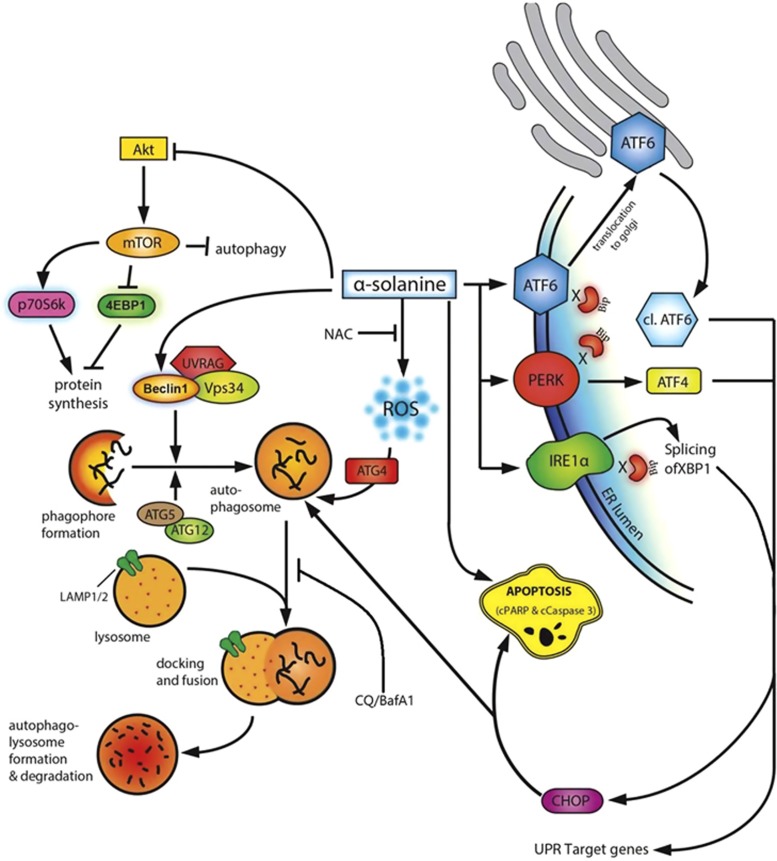
Proposed mechanism of *α*-solanine-induced cell death

In summary, this study demonstrates that *α*-solanine induces autophagy (in addition to apoptosis) to mediate cancer cell death. The GA was found to enhance intracellular ROS level and downregulate Akt/mTOR signaling pathway to activate autophagy. We also found that *α*-solanine triggers ER stress and activates UPR pathway to induce cell death. The findings of our study along with probable mode of action of *α*-solanine have been illustrated in Figure 8. Taken together, our results reiterate chemotherapeutic efficacy of *α*-solanine and provide first evidence of autophagy as an underlying mechanism.

## Materials and Methods

### Cell lines

A549 – human lung cancer cell, MCF-7 – human breast cancer cell, C33A – human cervical cancer cell, DU145 – human prostate cancer cell and KB – HeLa contaminant of human epidermoid carcinoma cell lines were obtained from American Type Culture Collection (ATCC, Manassas, VA, USA). Cells were maintained in Dulbecco's modified Eagle's medium (Sigma-Aldrich, St. Louis, MO, USA) supplemented with 10% fetal bovine serum (Gibco BRL, Gaithersburg, MD, USA) and antibiotic–antimycotic solution (Gibco BRL).

### Chemicals and antibodies

BafA1, *α*-solanine, CQ, doxorubicin,*N*-acetyl-L-cysteine (NAC), SRB sodium salt, AKT 1/2 kinase inhibitor, anti-LC3B and anti-*β* actin were purchased from Sigma-Aldrich. Rapamycin was procured from Millipore Corporation (Billerica, MA, USA). Anti-PARP, anti-Atg5, anti-phospho-Akt (Ser473), anti-4E-BP1, anti-phospho-mTOR (Ser2448), anti-phospho-4E-BP1 (Thr37/46), anti-caspase -3, anti-phospho p70 S6 kinase (Thr389), anti-phospho-Akt (Thr308), anti-Akt(pan), anti-mTOR, anti-phospho mTOR, anti-GRP78 (BiP), anti-IRE1*α*, anti-PERK, anti-CHOP and anti-LC3 were obtained from Cell Signaling Technology (Danvers, MA, USA). Paraformaldehyde, anti-ATF-6*α*, anti-XBP-1, anti-CREB-2, anti-GADD153/CHOP (B-3), anti-Beclin 1 and anti-LAMP-2 were procured from Santa Cruz Biotechnology (Santa Cruz, CA, USA). Anti-glyceraldehyde-3-phosphate dehydrogenase (GAPDH) was obtained from Imgenex (IMGENEX India Pvt. Ltd., Bhubaneswar, India). All fluorescence-conjugated and peroxidase-conjugated secondary antibodies were purchased from Invitrogen Corp., (Carlsbad, CA, USA) and Thermo Scientific (Rockford, IL, USA), respectively.

### Cell viability assay

Cytotoxic effect of *α*-solanine on different cancer cells was measured by SRB assay. Cells (10^4^ per well) were seeded onto 96-well plates and grown overnight before being treated with or without *α*-solanine at different concentrations. After 48- h incubation with *α*-solanine, cells were fixed and stained with SRB dye as described earlier.^66^ Bound dye was solubilized with 10 mM Tris base and the plates were read at 510 nm absorbance.

### Measurement of ROS and mitochondrial superoxide

Intracellular level of ROS was determined by fluorescence microscopy as well as by flow cytometry using CM-H_2_DCFDA (Invitrogen Corp.,). For microscopy, cells were grown overnight at 37 °C and treated with *α*-solanine for 24 h. Cells were then incubated with 10 *μ*M CM-H_2_DCFDA for 30 min in dark, washed with PBS and examined under confocal microscope. Similarly, A549 cells were trypsinized after incubation with or without *α*-solanine for 24 h. Cells (2x10^6^) were then resuspended in 500 *μ*l HBSS after a brief wash with PBS, stained with CM-H_2_DCFDA for 30 min in dark and analyzed by FACS Caliber flow cytometer (BD Biosciences, San Jose, CA, USA). To further confirm our finding in presence of ROS scavenger, cells were pre-incubated with NAC for 2 h before exposure to *α*-solanine.

Mitochondrial superoxide level in *α*-solanine-treated cells was investigated by confocal microscopy using MitoSOX Red dye (Invitrogen Molecular Probes, Eugene, OR, USA). Briefly, cells were grown overnight on coverslips, treated with *α*-solanine for 24 h and stained with 4 *μ*M MitoSOX Red dye for 10 min in dark. After washing with warm PBS, cells were observed under microscope.

### Determination of mitochondrial membrane potential (MMP) and release of cytochrome c

Integrity of mitochondrial outer membrane was investigated by staining cells with a cationic dye, JC1. Cells were grown overnight in confocal glass bottom dishes and treated with *α*-solanine for 24 h. Cells were then washed with PBS and stained with 2 *μ*M JC1 for 30 min. Samples were finally washed with PBS and examined under confocal microscope.

Release of cytochrome c from mitochondria was assessed by confocal microscopy. Briefly, cells were washed with PBS after incubation with *α*-solanine for 24 h, fixed with 4% paraformaldehyde and permeabilized with 0.5% Triton X100. Samples were then blocked with 2% BSA (in PBS) for 1 h and probed with anti-cytochrome c antibody for overnight at 4 °C. Cell were then stained with Alexa Fluor 488 conjugated secondary antibody for 90 min at room temperature (RT) and examined under microscope.

### Lipid peroxidation assay

Relative levels of lipid peroxidation were measured with cis-parinaric acid assay using standard protocol. Briefly, cells were seeded onto 96-well plates and treated accordingly. Cells were then washed once with warm PBS and incubated with 10 *μ*M cis-parinaric acid for 1 h at 37 °C. After washing with warm PBS, fluorescence intensities were measured in a spectrophotometer at 360 nm excitation and 460 nm emission.

### Estimation of cytosolic calcium levels

The experiment was performed as reported earlier with minor modifications.^39^ In brief, cells were grown in glass bottom confocal dishes overnight. The next day, cells were treated with either *α*-solanine (10 *μ*M, 24 h) or 0.5 *μ*M thapsigargin (TG; Calbiochem, San Diego, CA, USA) for 16 h. TG was used as positive control for ER calcium release. Cells were stained with 5 *μ*M Fluo-4AM (Invitrogen Molecular Probes) in calcium free Dulbecco's phosphate-buffered saline (DPBS) for 1 h before imaging.

### Scanning electron microscopy

Cellular morphology was studied using SEM. Cells grown on cover slips were fixed in 2.5% glutaraldehyde in 0.1 M phosphate buffer. After washing in phosphate buffer, samples were post-fixed in 1% OsO_4_ and subsequently dehydrated through an ascending ethanol series, critical point dried and coated with Au-Pd (80:20) using a Polaron E5000 sputter coater (Polaron Equipments Ltd, Hertfordshire, UK). Samples were examined in a FEI Quanta 250 SEM at an accelerating voltage of 15 kV using SE detector. About 200 cells from two stubs for each sample were analyzed.

### Transmission electron microscopy (TEM)

Ultrastructural alterations were analyzed by using TEM thin sectioning technique as reported earlier with minor modifications.^67^ In brief, cells were fixed with 4% paraformaldehyde (PFA) and 2% glutaraldehyde in 0.1 M phosphate buffer, pH 7.4 for 4 h at 4 °C. Samples were washed in 0.1 M phosphate buffer, post-fixed in 2% OsO_4_ at RT and encapsulated in agarose. This was followed by dehydration in ascending grades of ethanol, infiltration and embedding in Spurr Resin and polymerization at 60 °C for 24 h. Ultrathin sections (50–70 nm) were obtained using an ultramicrotome (Leica Ultracut UCT, Leica Microsystems GmbH, Wetzlar, Germany) and picked up onto 200 mesh copper grids. The sections were double stained with uranyl acetate and lead citrate and observed under a Jeol JEM 1400 Transmission Electron Microscope equipped with Gatan ES500 Erlangshen and Gatan Orius SC200B CCD cameras at 80kV (Gatan Inc., Pleasanton, CA, USA). At least 300 cells were analyzed from three independent experiments.

### Immunofluorescence staining

A549 cells were grown on coverslips for overnight and treated with *α*-solanine for indicated time periods. After removing culture media, cells were fixed with 4% PFA, permeabilized in 0.5% Triton X100 in PBS and blocked with 2% BSA. Subsequently, cells were probed overnight with primary antibody at 4 °C, washed thrice with PBS and incubated with corresponding fluorescence-conjugated secondary antibody for 1 h at RT. Images were acquired using a Carl Zeiss LSM 510 META confocal microscope (Carl Zeiss, Jena, Germany) equipped with a Plan Apochromat 63x oil/1.4 NA DIC objective.

### Western blot assay

Cells were lysed in buffer (25 mM HEPES, 0.4 M NaCl, 1.5 mM MgCl_2_, 0.2 mM EDTA, 1% NP 40) containing protease (Sigma-Aldrich) and phosphatase (Roche Diagnostics, Indianapolis, IN, USA) inhibitor for 1 h on ice. The cell lysates were centrifuged at 12000 *g* for 10 min and the protein content in supernatant was measured by BCA assay (Thermo Scientific). Equal amounts of protein were separated by SDS-PAGE and transferred onto PVDF membrane. Following overnight incubation with corresponding primary antibodies at 4 °C, membranes were washed and incubated with peroxidase conjugated secondary antibody for 1 h at RT. Specific protein bands were detected with an enhanced chemiluminescence reagent (Millipore Corporation) and visualized by a chemiluminescence detector (Bio-Rad Laboratories, Inc., Berkeley, CA, USA). The densitometric analysis of blots was done by Image J software (National Institutes of Health, Bethesda, MD, USA).

### Plasmid, siRNA and transfection

EGPF-LC3 plasmid was donated by Dr. Karla Kirkegaard (Addgene plasmid 11546; Cambridge, MA, USA).^68^ The siRNA targeting human *Beclin1*^(ref. 69)^ and scrambled control siRNA were obtained from Dharmacon, Inc., (Lafayette, CO, USA). Human *EIF2AK3* (PERK)-specific siRNA was procured from Qiagen Inc., (cat # SI02223718; Valencia, CA, USA). Cells were transfected with Lipofectamine 2000 (Invitrogen Corp.,) as per standard protocol and cultured for 48 h in complete medium before further analysis. The extent of gene knockdown was determined by immunoblotting.

To establish a stable C33A cell line expressing GFP-LC3, G418 (300 *μ*g/ml) was added to the culture media at 48 h after transfection with GFP-LC3 plasmid. Cells were then allowed to grow for 2 weeks in presence of G418 and viable stable clones were selected and propagated for further experiment.

### Statistical analysis

The statistical significance of the differences between two experimental groups from three independent experiments was assessed using two-tailed Student's *t*-test. A value of *P*<0.05 was considered statistically significant.

## Figures and Tables

**Table 1:** IC_50_ values of *α*-solanine in human cancer cells

	**IC_50_ (μM) (mean±S.E.)^a^**			
	**DU145**	**A549**	**KB**	**MCF-7**
*α*-Solanine	9.63±0.24	9.37±0.37	11.99±1.68	11.63±1.85

a *n*=4

## Abbreviations

ATF: activating transcription factor
CQ: chloroquine
XBP1: X-box-binding protein 1
LC3: microtubule-associated protein 1 (MAP1) light chain-3
mTOR: mammalian target of rapamycin
UPR: unfolded protein response
NAC: *N*-acetyl-L-cysteine
PARP: poly (ADP-ribose) polymerase
ATG: autophagy-related
CM-H_2_DCFDA: 5 (and 6)-chloromethyl-2′,7′-dichlorodihydrofluorescein diacetate acetyl ester
ROS: reactive oxygen species
SRB: sulforhodamine B
BafA1: bafilomycin A1
LAMP: lysosome-associated membrane protein
4E-BP1: eukaryotic initiation factor 4E-binding protein 1
IRE1: inositol-requiring transmembrane kinase/endonuclease 1
CHOP: CCAAT-enhancer-binding protein (C/EBP)-homologous protein
GAPDH: glyceraldehyde-3-phosphate dehydrogenase

## References

[bib1] Korpan YI Nazarenko EA Skryshevskaya IV Martelet C Jaffrezic-Renault N El'skaya AV Potato glycoalkaloids: true safety or false sense of security Trends Biotechnol 2004 22 147 151 15036866 10.1016/j.tibtech.2004.01.009

[bib2] Maga JA Potato glycoalkaloids Crit Rev Food Sci Nutr 1980 12 371 405 6996922 10.1080/10408398009527281

[bib3] Friedman M McDonald GM Filadelfi-Keszi M Potato glycoalkaloids: chemistry, analysis, safety, and plant physiology Crit Rev Plant Sci 1997 16 55 132

[bib4] McMillan M Thompson JC An outbreak of suspected solanine poisoning in schoolboys: examinations of criteria of solanine poisoning Q J Med 1979 48 227 243 504549

[bib5] Rayburn JR Bantle JA Friedman M Role of carbohydrate side chains of potato glycoalkaloids in developmental toxicity J Agr Food Chem 1994 42 1511 1515

[bib6] Kuiper-Goodman T Nawrot P Solanine and Chaconine World Health Organization: : Geneva, Switzerland 1993

[bib7] Nema PK Ramayya N Duncan E Niranjan K Potato glycoalkaloids: formation and strategies for mitigation J Sci Food Agr 2008 88 1869 1881

[bib8] Fewell AM Roddick JG Potato glycoalkaloid impairment of fungal development Mycol Res 1997 101 597 603

[bib9] Golubeva SN [Experiences in the diagnosis of food allergy and it's treatment with solanine] Vestnik otorinolaringologii 1966 28 23 27 6003813

[bib10] Delporte Vergara C Backhouse Erazo N Negrete R Salinas P Rivas Rubio PA Cassels BK Antipyretic, hypothermic and antiinflammatory activities and metabolites from *Solanum ligustrinum* Lood Phytother Res 1998 12 118 122

[bib11] Choi E Koo S Anti-nociceptive and anti-inflammatory effects of the ethanolic extract of potato (*Solanum**tuberlosum* Food Agr Immunol 2005 16 29 39

[bib12] Sato T Glycemic effects of solanine in rats Jpn J Pharmacol 1967 17 652 658 4385229

[bib13] Gubarev MI Enioutina EY Taylor JL Visic DM Daynes RA Plant derived glycoalkaloids protect mice against lethal infection with *Salmonella typhimurium* Phytother Res 1998 12 79 88

[bib14] Paquin R Lachance RA [Effect of glycoalcaloids of the potato on the growth of Corynebacterium Sepedonicum (Spieck. And Kott.) Skapt. And Burkh] Can J Microbiol 1964 10 115 122 14171638 10.1139/m64-018

[bib15] Ikeda T Ando J Miyazono A Zhu XH Tsumagari H Nohara T Anti-herpes virus activity of *Solanum* steroidal glycosides Biol Pharm Bull 2000 23 363 364 10726897 10.1248/bpb.23.363

[bib16] Thorne HV Clarke GF Skuce R The inactivation of herpes simplex virus by some *Solanaceae* glycoalkaloids Antiviral Res 1985 5 335 343 3004327 10.1016/0166-3542(85)90003-8

[bib17] Giron LM Aguilar GA Caceres A Arroyo GL Anticandidal activity of plants used for the treatment of vaginitis in Guatemala and clinical trial of a *Solanum nigrescens* preparation J Ethnopharmacol 1988 22 307 313 3292844 10.1016/0378-8741(88)90241-3

[bib18] Chataing B Concepcion JL Lobaton R Usubillaga A Inhibition of Trypanosoma cruzi growth *in vitro* by *Solanum* alkaloids: a comparison with ketoconazole Planta Medica 1998 64 31 36 9491766 10.1055/s-2006-957361

[bib19] Friedman M Lee KR Kim HJ Lee IS Kozukue N Anticarcinogenic effects of glycoalkaloids from potatoes against human cervical, liver, lymphoma, and stomach cancer cells J Agr Food Chem 2005 53 6162 6169 16029012 10.1021/jf050620p

[bib20] Lee KR Kozukue N Han JS Park JH Chang EY Baek EJ Glycoalkaloids and metabolites inhibit the growth of human colon (HT29) and liver (HepG2) cancer cells J Agr Food Chem 2004 52 2832 2839 15137822 10.1021/jf030526d

[bib21] Lu MK Shih YW Chang Chien TT Fang LH Huang HC Chen PS Alpha-Solanine inhibits human melanoma cell migration and invasion by reducing matrix metalloproteinase-2/9 activities Biol Pharm Bull 2010 33 1685 1691 20930376 10.1248/bpb.33.1685

[bib22] Lv C Kong H Dong G Liu L Tong K Sun H Antitumor efficacy of alpha-solanine against pancreatic cancer *in vitro* and *in vivo* PloS One 2014 9 e87868 24505326 10.1371/journal.pone.0087868 PMC3914882

[bib23] Mohsenikia M Alizadeh AM Khodayari S Khodayari H Kouhpayeh SA Karimi A The protective and therapeutic effects of alpha-solanine on mice breast cancer Eur J Pharmacol 2013 718 1 9 24051269 10.1016/j.ejphar.2013.09.015

[bib24] Sun H Lv C Yang L Wang Y Zhang Q Yu S Solanine induces mitochondria-mediated apoptosis in human pancreatic cancer cells BioMed Res Int 2014 2014 805926 24949471 10.1155/2014/805926 PMC4037623

[bib25] He C Klionsky DJ Regulation mechanisms and signaling pathways of autophagy Ann Rev Genet 2009 43 67 93 19653858 10.1146/annurev-genet-102808-114910 PMC2831538

[bib26] Levine B Klionsky DJ Development by self-digestion: molecular mechanisms and biological functions of autophagy Dev Cell 2004 6 463 477 15068787 10.1016/s1534-5807(04)00099-1

[bib27] Kimmelman AC The dynamic nature of autophagy in cancer Genes Dev 2011 25 1999 2010 21979913 10.1101/gad.17558811 PMC3197199

[bib28] Ravikumar B Sarkar S Davies JE Futter M Garcia-Arencibia M Green-Thompson ZW Regulation of mammalian autophagy in physiology and pathophysiology Physiol Rev 2010 90 1383 1435 20959619 10.1152/physrev.00030.2009

[bib29] Janku F McConkey DJ Hong DS Kurzrock R Autophagy as a target for anticancer therapy Nat Rev Clin Oncol 2011 8 528 539 21587219 10.1038/nrclinonc.2011.71

[bib30] Kroemer G Jaattela M Lysosomes and autophagy in cell death control Nat Rev Cancer 2005 5 886 897 16239905 10.1038/nrc1738

[bib31] Li H Wang P Sun Q Ding WX Yin XM Sobol RW Following cytochrome c release, autophagy is inhibited during chemotherapy-induced apoptosis by caspase 8-mediated cleavage of Beclin 1 Cancer Res 2011 71 3625 3634 21444671 10.1158/0008-5472.CAN-10-4475 PMC3096685

[bib32] Chen S Rehman SK Zhang W Wen A Yao L Zhang J Autophagy is a therapeutic target in anticancer drug resistance Biochimica et Biophysica Acta 2010 1806 220 229 20637264 10.1016/j.bbcan.2010.07.003

[bib33] Tai CJ Wang CK Tai CJ Lin YF Lin CS Jian JY Aqueous extract of *Solanum nigrum* Leaves induces autophagy and enhances cytotoxicity of cisplatin, doxorubicin, docetaxel, and 5-fluorouracil in human colorectal carcinoma cells Evid Based Complementary Altern Med 2013 2013 514719 10.1155/2013/514719 PMC3703357 23843876

[bib34] Mann SS Hammarback JA Molecular characterization of light chain 3. A microtubule binding subunit of MAP1A and MAP1B J Biol Chem 1994 269 11492 11497 7908909

[bib35] Kabeya Y Mizushima N Ueno T Yamamoto A Kirisako T Noda T LC3, a mammalian homologue of yeast Apg8p, is localized in autophagosome membranes after processing EMBO J 2000 19 5720 5728 11060023 10.1093/emboj/19.21.5720 PMC305793

[bib36] Kabeya Y Mizushima N Yamamoto A Oshitani-Okamoto S Ohsumi Y Yoshimori T LC3, GABARAP and GATE16 localize to autophagosomal membrane depending on form-II formation J Cell Sci 2004 117 2805 2812 15169837 10.1242/jcs.01131

[bib37] Mizushima N Yoshimori T Levine B Methods in mammalian autophagy research Cell 2010 140 313 326 20144757 10.1016/j.cell.2010.01.028 PMC2852113

[bib38] Scherz-Shouval R Shvets E Fass E Shorer H Gil L Elazar Z Reactive oxygen species are essential for autophagy and specifically regulate the activity of Atg4 EMBO J 2007 26 1749 1760 17347651 10.1038/sj.emboj.7601623 PMC1847657

[bib39] Abdelrahim M Newman K Vanderlaag K Samudio I Safe S 3,3'-diindolylmethane (DIM) and its derivatives induce apoptosis in pancreatic cancer cells through endoplasmic reticulum stress-dependent upregulation of DR5 Carcinogenesis 2006 27 717 728 16332727 10.1093/carcin/bgi270

[bib40] Ji YB Gao SY Ji CF Zou X Induction of apoptosis in HepG2 cells by solanine and Bcl-2 protein J Ethnopharmacol 2008 115 194 202 18022776 10.1016/j.jep.2007.09.023

[bib41] Ouyang DY Xu LH He XH Zhang YT Zeng LH Cai JY Autophagy is differentially induced in prostate cancer LNCaP, DU145 and PC-3 cells via distinct splicing profiles of ATG5 Autophagy 2013 9 20 32 23075929 10.4161/auto.22397 PMC3542215

[bib42] Saiki S Sasazawa Y Imamichi Y Kawajiri S Fujimaki T Tanida I Caffeine induces apoptosis by enhancement of autophagy via PI3K/Akt/mTOR/p70S6K inhibition Autophagy 2011 7 176 187 21081844 10.4161/auto.7.2.14074 PMC3039768

[bib43] Djavaheri-Mergny M Maiuri MC Kroemer G Cross talk between apoptosis and autophagy by caspase-mediated cleavage of Beclin 1 Oncogene 2010 29 1717 1719 20101204 10.1038/onc.2009.519

[bib44] Yousefi S Perozzo R Schmid I Ziemiecki A Schaffner T Scapozza L Calpain-mediated cleavage of Atg5 switches autophagy to apoptosis Nat Cell Biol 2006 8 1124 1132 16998475 10.1038/ncb1482

[bib45] Ding WX Ni HM Gao W Hou YF Melan MA Chen X Differential effects of endoplasmic reticulum stress-induced autophagy on cell survival J Biol Chem 2007 282 4702 4710 17135238 10.1074/jbc.M609267200

[bib46] Ogata M Hino S Saito A Morikawa K Kondo S Kanemoto S Autophagy is activated for cell survival after endoplasmic reticulum stress Mol Cell Biol 2006 26 9220 9231 17030611 10.1128/MCB.01453-06 PMC1698520

[bib47] Yorimitsu T Klionsky DJ Endoplasmic reticulum stress: a new pathway to induce autophagy Autophagy 2007 3 160 162 17204854 10.4161/auto.3653

[bib48] Ron D Walter P Signal integration in the endoplasmic reticulum unfolded protein response Nat Rev Mol Cell Biol 2007 8 519 529 17565364 10.1038/nrm2199

[bib49] Walter P Ron D The unfolded protein response: from stress pathway to homeostatic regulation Science 2011 334 1081 1086 22116877 10.1126/science.1209038

[bib50] Margariti A Li H Chen T Martin D Vizcay-Barrena G Alam S XBP1 mRNA splicing triggers an autophagic response in endothelial cells through BECLIN-1 transcriptional activation J Biol Chem 2013 288 859 872 23184933 10.1074/jbc.M112.412783 PMC3543035

[bib51] Xu C Bailly-Maitre B Reed JC Endoplasmic reticulum stress: cell life and death decisions J Clin Invest 2005 115 2656 2664 16200199 10.1172/JCI26373 PMC1236697

[bib52] Clarke PG Developmental cell death: morphological diversity and multiple mechanisms Anat Embryol 1990 181 195 213 2186664 10.1007/BF00174615

[bib53] Hetz C The unfolded protein response: controlling cell fate decisions under ER stress and beyond Nat Rev Mol Cell Biol 2012 13 89 102 22251901 10.1038/nrm3270

[bib54] Harding HP Zhang Y Zeng H Novoa I Lu PD Calfon M An integrated stress response regulates amino acid metabolism and resistance to oxidative stress Mol Cell 2003 11 619 633 12667446 10.1016/s1097-2765(03)00105-9

[bib55] Dudek H Datta SR Franke TF Birnbaum MJ Yao R Cooper GM Regulation of neuronal survival by the serine-threonine protein kinase Akt Science 1997 275 661 665 9005851 10.1126/science.275.5300.661

[bib56] Noda T Ohsumi Y Tor, a phosphatidylinositol kinase homologue, controls autophagy in yeast J Biol Chem 1998 273 3963 3966 9461583 10.1074/jbc.273.7.3963

[bib57] Gibbons JJ Abraham RT Yu K Mammalian target of rapamycin: discovery of rapamycin reveals a signaling pathway important for normal and cancer cell growth Semin Oncol 2009 36 (Suppl 3 S3 S17 19963098 10.1053/j.seminoncol.2009.10.011

[bib58] Vander Haar E Lee SI Bandhakavi S Griffin TJ Kim DH Insulin signalling to mTOR mediated by the Akt/PKB substrate PRAS40 Nat Cell Biol 2007 9 316 323 17277771 10.1038/ncb1547

[bib59] Nave BT Ouwens M Withers DJ Alessi DR Shepherd PR Mammalian target of rapamycin is a direct target for protein kinase B: identification of a convergence point for opposing effects of insulin and amino-acid deficiency on protein translation Biochem J 1999 344 (Pt 2 427 431 10567225 PMC1220660

[bib60] Peterson RT Beal PA Comb MJ Schreiber SL FKBP12-rapamycin-associated protein (FRAP) autophosphorylates at serine 2481 under translationally repressive conditions J Biol Chem 2000 275 7416 7423 10702316 10.1074/jbc.275.10.7416

[bib61] Copp J Manning G Hunter T TORC-specific phosphorylation of mammalian target of rapamycin (mTOR): phospho-Ser2481 is a marker for intact mTOR signaling complex 2 Cancer Res 2009 69 1821 1827 19244117 10.1158/0008-5472.CAN-08-3014 PMC2652681

[bib62] Chang YY Neufeld TP An Atg1/Atg13 complex with multiple roles in TOR-mediated autophagy regulation Mol Biol Cell 2009 20 2004 2014 19225150 10.1091/mbc.E08-12-1250 PMC2663935

[bib63] Ganley IG Lam du H Wang J Ding X Chen S Jiang X ULK1.ATG13.FIP200 complex mediates mTOR signaling and is essential for autophagy J Biol Chem 2009 284 12297 12305 19258318 10.1074/jbc.M900573200 PMC2673298

[bib64] Hosokawa N Hara T Kaizuka T Kishi C Takamura A Miura Y Nutrient-dependent mTORC1 association with the ULK1-Atg13-FIP200 complex required for autophagy Mol Biol Cell 2009 20 1981 1991 19211835 10.1091/mbc.E08-12-1248 PMC2663915

[bib65] Sarbassov DD Guertin DA Ali SM Sabatini DM Phosphorylation and regulation of Akt/PKB by the rictor-mTOR complex Science 2005 307 1098 1101 15718470 10.1126/science.1106148

[bib66] Adaramoye OA Sarkar J Singh N Meena S Changkija B Yadav PP Antiproliferative action of Xylopia aethiopica fruit extract on human cervical cancer cells Phytother Res 2011 25 1558 1563 21698670 10.1002/ptr.3551

[bib67] Kathuria M Bhattacharjee A Sashidhara KV Singh SP Mitra K Induction of mitochondrial dysfunction and oxidative stress in Leishmania donovani by orally active clerodane diterpene Antimicrob Agents Chemother 2014 58 5916 5928 25070112 10.1128/AAC.02459-14 PMC4187897

[bib68] Jackson WT Giddings TH Jr. Taylor MP Mulinyawe S Rabinovitch M Kopito RR Subversion of cellular autophagosomal machinery by RNA viruses PLoS Biol 2005 3 e156 15884975 10.1371/journal.pbio.0030156 PMC1084330

[bib69] Fremont S Gerard A Galloux M Janvier K Karess RE Berlioz-Torrent C Beclin-1 is required for chromosome congression and proper outer kinetochore assembly EMBO Rep 2013 14 364 372 23478334 10.1038/embor.2013.23 PMC3615652

