# Supplementary figures and images for: α-Solanine induces ROS-mediated autophagy through activation of endoplasmic reticulum stress and inhibition of Akt/mTOR pathway
Source: Cell Death Dis. 2015 Aug 27;6(8):e1860–. doi: 10.1038/cddis.2015.219 (PMC4558510; doi:10.1038/cddis.2015.219)

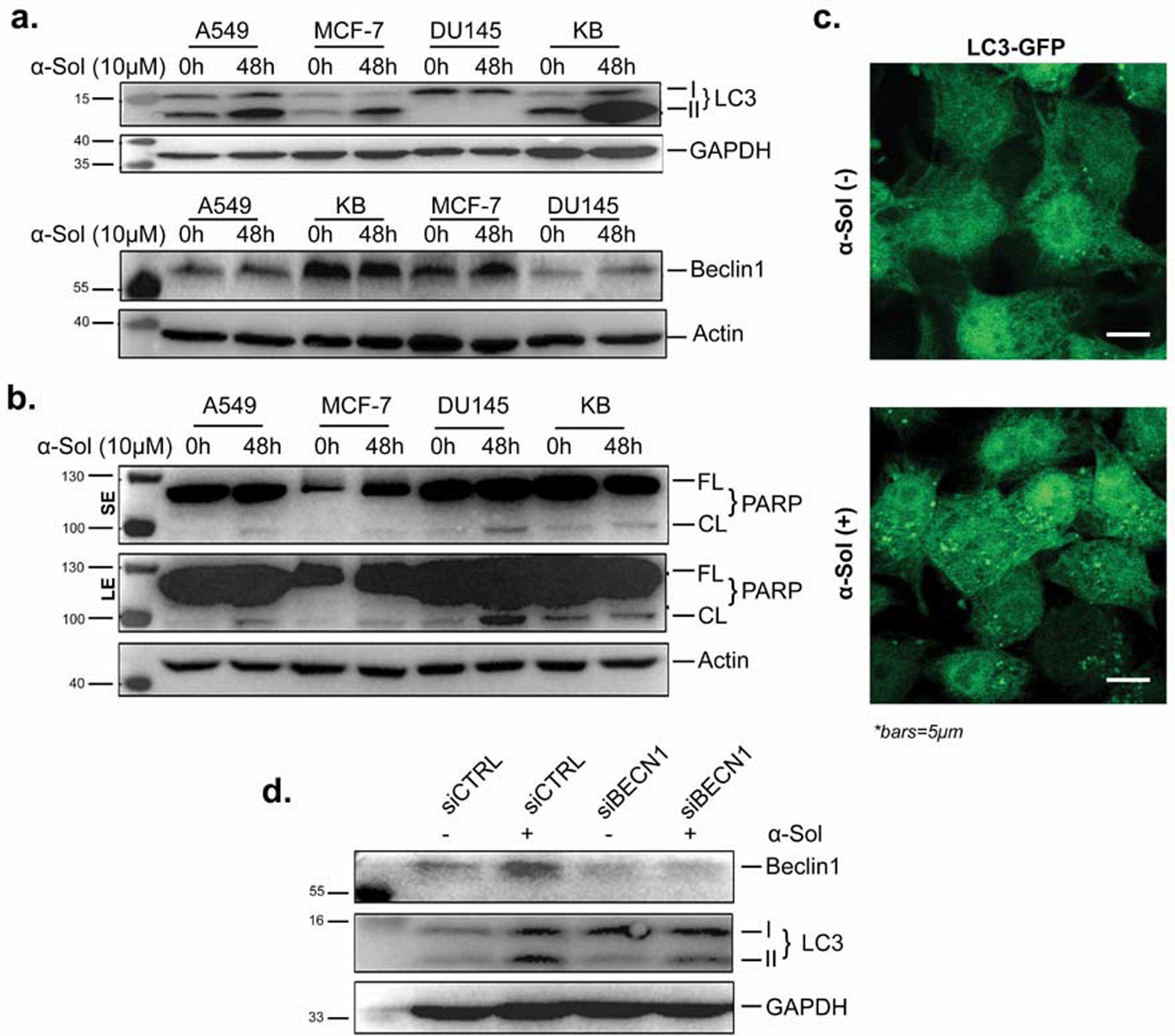

Supplement: Supplementary Figure 1 [file cddis2015219x1.tif]

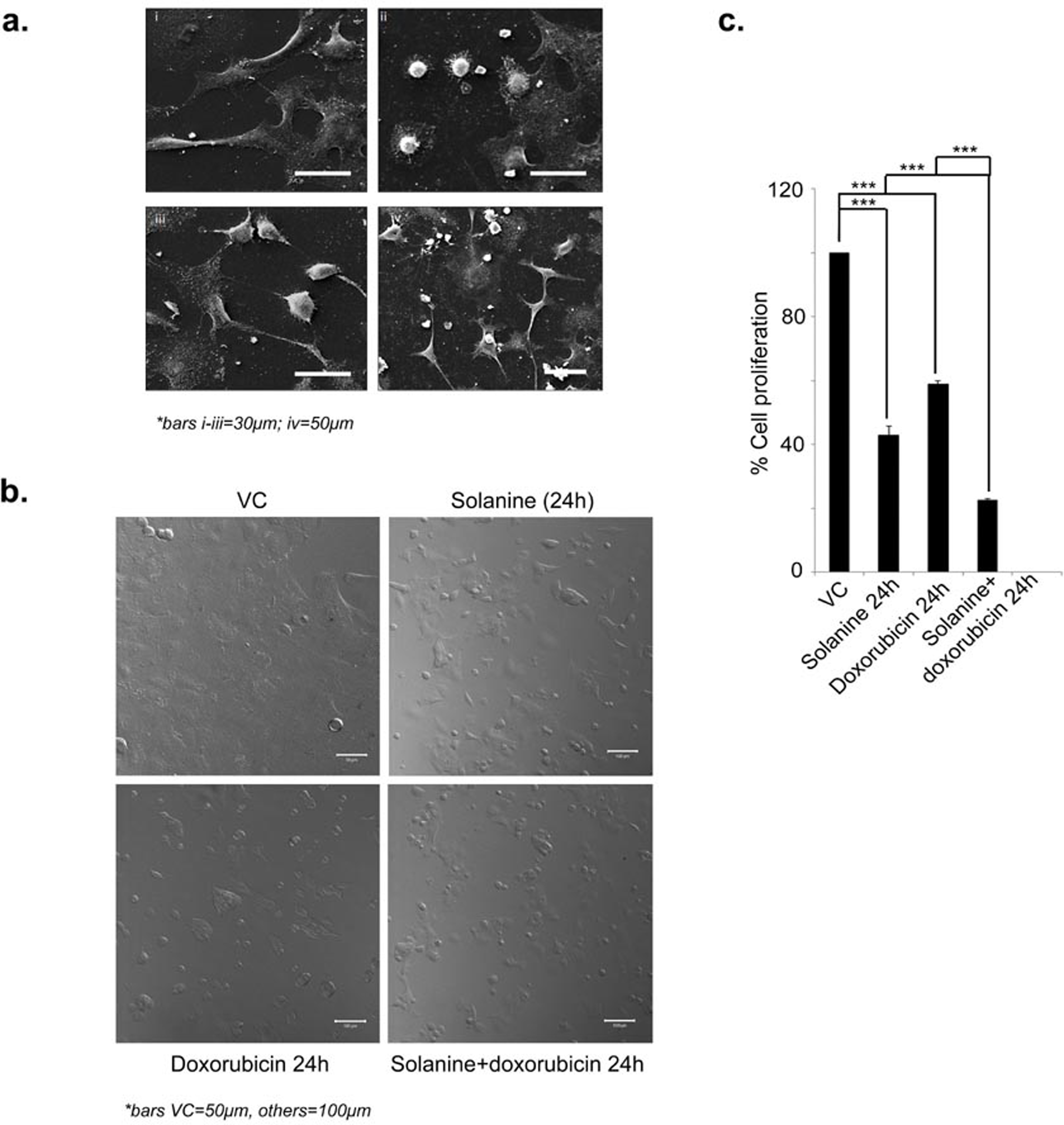

Supplement: Supplementary Figure 2 [file cddis2015219x2.tif]

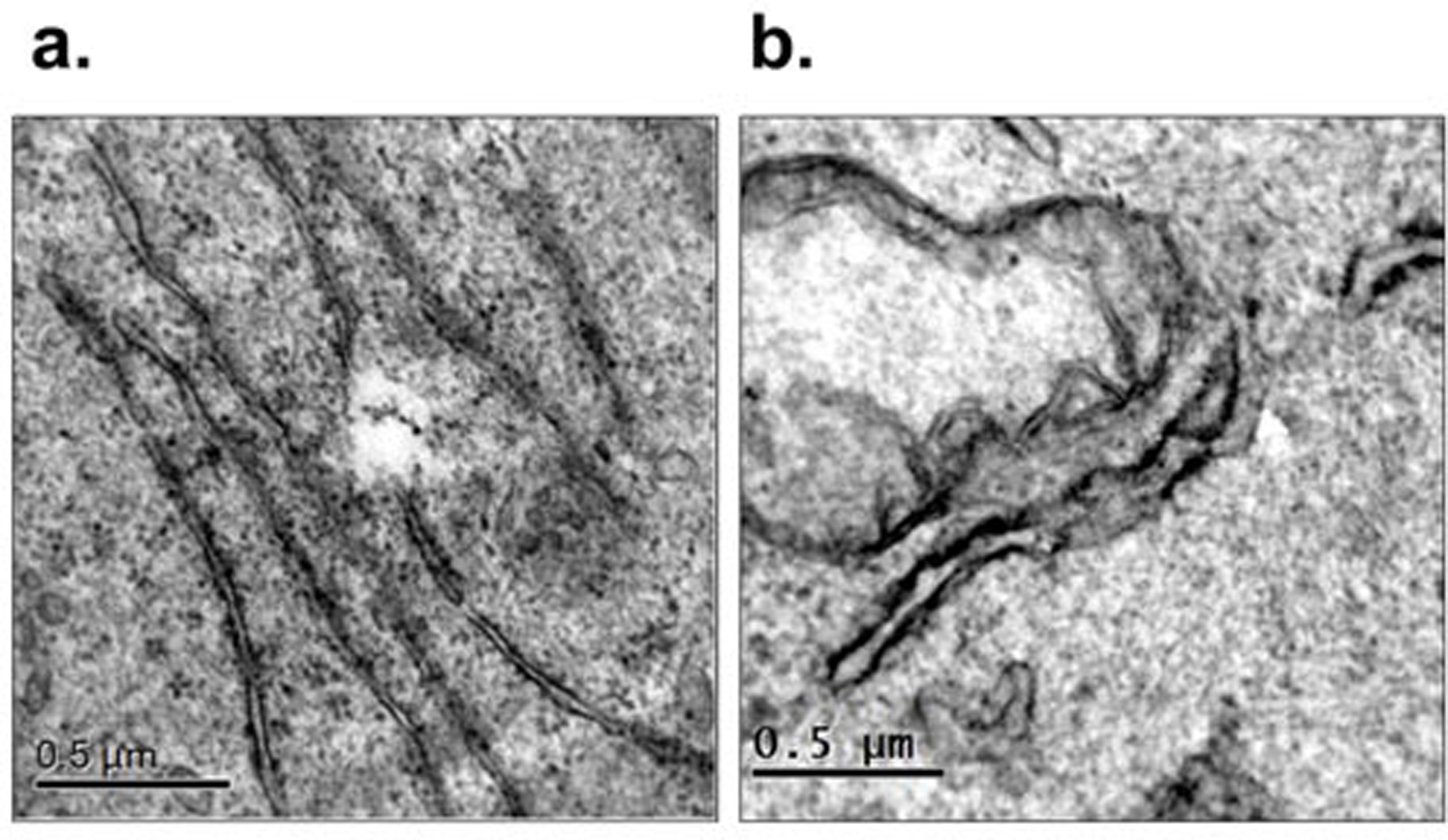

Supplement: Supplementary Figure 3 [file cddis2015219x3.tif]

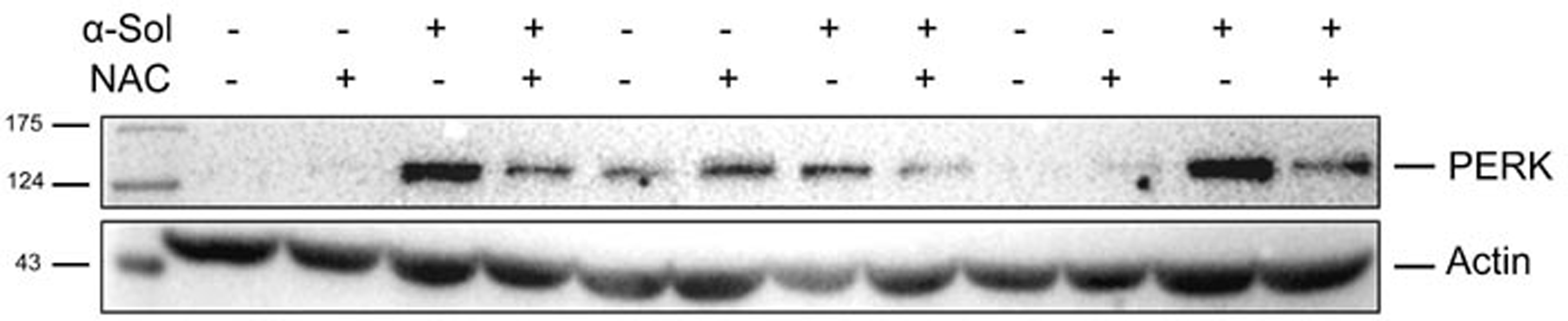

Supplement: Supplementary Figure 4 [file cddis2015219x4.tif]
