# Supplementary material for: α-Solanine induces ROS-mediated autophagy through activation of endoplasmic reticulum stress and inhibition of Akt/mTOR pathway
Source: Cell Death Dis. 2015 Aug 27;6(8):e1860–. doi: 10.1038/cddis.2015.219 (PMC4558510; doi:10.1038/cddis.2015.219)
Supplement: Supplementary Figure Legends [file cddis2015219x5.doc]

Figure S1:

α-Solanine induced both autophagy and apoptosis in human cancer cells. (A) Cells were treated with or without α-solanine for 48 h. Cell lysates were analyzed by immunoblotting for induction of autophagy. (B) Induction of apoptosis in different human cancer cell lines was determined by measuring PARP cleavage by immunoblot assay after 48 h α-solanine treatment. LE, long exposure; SE, short exposure. (C) C33A cells stably expressing GFP-LC3 were treated with or without 10 µM α-solanine for 24 h and analyzed under confocal microscope for accumulation of puncta. (D) A549 cells were transfected with scrambled and siRNA against Beclin1 and exposed to 10 µM α-solanine for 24 h. The level of autophagy was determined by immunoblotting of lipid conjugated LC-3.

Figure S2:

α-Solanine treatment caused additionally enhanced cytotoxicity of doxorubicin. A459 cells were treated with 4 µM doxorubicin and 10 µM α-solanine for indicated time hours. (A) SEM micrographs showing i) control A549 cells; ii) 4µM doxorubicin treated A549 cells for 24h; iii) 10µM α- solanine treated A549 cells for 24h; iv) 4µM doxorubicin + 10µM α- solanine treated A549 cells for 24h. Enhanced cytotoxic effect was observed (iv) when the cells were co-treated with α-solanine and doxorubicin. Note the round apoptotic cells in ii & iv exhibiting blebbing and apoptotic bodies. Control cells (i) show normal spindle shaped morphology while α-solanine treated cells exhibit minor swelling without apoptosis. (B) Morphology of the cells was captured by phase contrast microscope (Nikon TiE). (C) Cell viability was measured by SRB assay. Data presented as means ± SE. *** *P* <0.005.

Figure S3:

α-Solanine induced ER stress in A549 cells. TEM micrographs showing rough ER in vehicle treated A549 cells (A) and smooth swollen ER after incubation with α-solanine (B).

Figure S4:

α-Solanine induced ER stress was ROS dependent. A549 cells lysates, treated with 10 µM α-solanine in presence or absence of 5 mM NAC, were analyzed for PERK expression by Western blot assay.
